# Psychological Distress Among Chinese Manufacturing Employees: Prevalence and a Symptom Network Analysis

**DOI:** 10.1002/pchj.70015

**Published:** 2025-04-29

**Authors:** Jie Meng, Xueping Meng

**Affiliations:** ^1^ Department of Psychology, Faculty of Education Guangxi Normal University Guilin China; ^2^ Key Laboratory of Cognitive Neuroscience and Applied Psychology (Guangxi Normal University), Education Department of Guangxi Zhuang Autonomous Region Guilin China

**Keywords:** anxiety, burnout, depression, manufacturing employees, network analysis

## Abstract

The psychological distress among manufacturing workers is an increasingly important issue and has attracted extensive attention. However, the mental health of this subgroup of the Chinese population is underexplored. This study aimed to evaluate the prevalence of psychological distress in Chinese manufacturing employees and identify central symptoms, important bridge symptoms, and associations between symptoms using network analysis. The participants were 4934 employees recruited from a Chinese manufacturing company. The Maslach Burnout Inventory‐General Survey (MBI‐GS), the Self‐Rating Anxiety Scale (SAS), the Self‐Rating Depression Scale (SDS), and the Symptom Checklist 90 (SCL‐90) were used to assess job burnout, anxiety, depression, compulsive symptom, somatization, psychoticism, paranoid, phobic, hostility, and interpersonal sensitivity, respectively. In total, 29.77%, 21.14%, and 26.53% of all participants experienced burnout, anxiety, and depression, respectively. Compared to normative data of the Chinese population, the seven symptoms of the SCL‐90 among participants were significantly higher. The network analysis revealed that interpersonal sensitivity had the greatest strength and somatization had the greatest betweenness and closeness. Anxiety had the highest bridge expected influence. These results demonstrate that the mental health of Chinese manufacturing employees is a cause for concern. Interpersonal sensitivity and somatization emerged as the core symptoms, and anxiety was an important bridge symptom. Interventions aimed at these conditions may promote and enhance the overall mental health of Chinese manufacturing employees.

## 1 Introduction

Mental health issues among manufacturing employees have become more prevalent in recent years. Manufacturing employees are not strictly defined by company size or region and encompass various roles within manufacturing organizations, including managers, production managers, production workers, support staff, research and development/engineering (R&D/engineering) and sales (Hunt et al. 2015). A variety of factors in manufacturing industries, including long work hours, lack of support, and extreme stress, can lead to an increased risk of mental health issues among workers (Prasad 2023). It has been discovered that working in manufacturing industries could have a significant impact on a person's well‐being and mental health (Hulls et al. 2020). Facing an increasing amount of competition in the market, industrial workers are exposed to harsh work conditions and long hours in order to increase productivity. Additionally, manufacturing industries utilize more and more automation and robotics in recent years causing a reduction in manual labor, which could lead to a higher level of stress among industrial workers (Prasad 2023). A systematic review of mental health risk factors in manufacturing sectors revealed that the main risk factors at workplace were job expectations, task overload, unsupportive workplace relationships, and poor health and lifestyles choices, which result in the higher rates of depression and anxiety that industrial workers may experience (Battams et al. 2014). Similarly, a systematic review about employees' mental health status including several nations (Australia, Scotland, Japan, France, Canada, and the United States) showed that employees in manufacturing industries had depression levels that were much higher than the national average, suggesting that the higher rates of depression in the industrial group were consistent across different cultures (Roche et al. 2016).

Though the fact that employees in the manufacturing industry are vulnerable to poor mental health has been confirmed, manufacturing administrators often fail to provide adequate psychological intervention and assistance to the employees (Prasad 2023). There are a number of workers in manufacturing industries, and the lack of mental health services may lead to more distress among workers and further disruption in society. In 2023, the number of employees in China's manufacturing industry reached 104.713 million, ranking first among all industries (Chinese National Data 2024). However, the mental health status of this subgroup of the Chinese population is underexplored, which means that a substantial number of Chinese manufacturing workers may be at risk of experiencing deep psychological strain or tension, and may need psychological intervention and assistance. Evidence suggests that due to various risk factors, such as work involving minimal contact with others, excessive or irregular workload, and monotonous tasks, employees worked in manufacturing industries are more likely to experience psychiatric problems or mental issues, such as anxiety and depression (Roche et al. 2016). The mental health status of employees is important in the workplace since it is closely related to employees' wellbeing and productivity. The lack of available information about the mental health status of Chinese manufacturing employees makes it more challenging for company administrators and mental healthcare providers to offer targeted health assistance. Thus, this study aimed to fill this gap.

Depression and anxiety are common psychological problems, and workers in the manufacturing workforce are more likely to struggle with depression and anxiety (Prasad 2023). The prevalence of depression and anxiety among industrial workers varies across studies but is generally higher than national averages. A study showed that 15.2% of Korean female manufacturing workers experienced anxiety symptoms (Lee et al. 2015). Another study in India observed a 36% prevalence of anxiety among factory workers (Rao and Ramesh 2015). Similarly, a meta‐analysis found an overall prevalence of 21% in industrial workers, and prevalence rates differ by region, with Asia (22%) showing higher rates than Europe (18%) and America (20%) (Amiri 2022). Besides, job burnout is a significant concern across various sectors, with manufacturing and industrial workers particularly affected (Maslach et al. 2001). It is defined as a symptom emerging from continuous occupational stress that is not properly handled in the International Classification of Diseases, Eleventh Edition (ICD‐11, World Health Organization 2024). Studies have found high prevalence rates of burnout in the industrial sector. One study revealed that 25% of industrial workers had severe burnout, while 69.3% experienced moderate burnout (Pargov and Stoyanova 2023). Another study revealed that in Mexico, the overall prevalence of burnout was 15.9%, with operating‐level workers showing higher rates of 17.4% (Martínez‐Mejía et al. 2020). Besides, research has indicated that burnout has been associated with certain diseases, such as physical ailments (Brandstätter et al. 2016; Salvagioni et al. 2017), depression (Ahola et al. 2005; Bianchi et al. 2014), and anxiety (Mousavi et al. 2017). Since burnout is common among today's workers and is harmful to those who experience it as well as society as a whole, it has received a lot of attention. Additionally, the Symptom Checklist‐90 (SCL‐90) is a widely used self‐report inventory measuring psychological distress across nine symptom dimensions, which is widely used in China (Tian et al. 2020). Studies have applied the SCL‐90 to assess mental health in various populations, including manufacturing workers. Research on Chinese female workers found a 34.89% positive rate, with obsessive‐compulsive symptoms, somatization, and depression being the most prevalent issues (Wang et al. 2017). A Korean study of textile industry workers revealed higher symptom scores among manual laborers, with obsessive‐compulsive symptoms ranking highest (Choi 1982).

Given the prevalence of the psychological symptoms mentioned above and their detrimental effect on employees' overall health and productivity, this study aimed to assess the existence and prevalence of depression, anxiety, burnout, and symptoms of SCL‐90 experienced by Chinese manufacturing employees. Furthermore, we used network analysis, a new approach to analyzing data in psychology and psychiatry, to explore the correlations and central symptoms among these adverse psychological symptoms.

In recent years, network analysis offers a novel approach to understanding mental health constructs by conceptualizing symptoms as interconnected elements rather than reflections of underlying disorders (McNally 2016). This method provides more nuanced insights compared to traditional variable‐centered approaches by mapping complex associations among psychological variables (Briganti et al. 2024). In the network approach model, nodes represent the observed psychological symptoms and edges represent the partial connections among symptoms. In addition, through indicators (betweenness, closeness, strength, and bridge expected influence), this method enables researchers to pinpoint central and bridge symptoms that have a high correlation with numerous other symptoms. Owing to its high level of interconnectedness, the central and bridge symptoms are more likely than other symptoms to have a greater effect on the entire network (Beard et al. 2016).

Network analysis is based on the belief that all mental diseases result from the causal interactions between psychological symptoms, which are causally related to each other in a network, and these causal connections may be established based on biological processes, psychological systems, homeostatic combinations of factors, or societal values (Borsboom 2017). It allows for the examination of causal interactions between symptoms, potentially informing more effective intervention strategies (Wang and Cheng 2024). Besides, network analysis can be applied to various data structures, including cross‐sectional, repeated measures, and intensive longitudinal data (Borsboom et al. 2021). The approach has shown promise in transforming our understanding of psychopathology by revealing the intricate relationships between symptoms and their role in maintaining mental disorders (McNally 2016). Network analysis has been used to explore mental health in some Chinese risky groups, such as nurses (Wu et al. 2021) and medical undergraduates (Peng et al. 2023). Compared to conventional techniques of analysis, utilizing network analysis to explore the correlations among these psychological symptoms experienced by Chinese manufacturing employees will allow us to gain a deeper understanding of which symptoms might be central to this subset of the population.

Therefore, this study aims to (1) determine the prevalence of burnout and common psychological symptoms in Chinese manufacturing employees, and (2) map out the network of interrelationships among these symptoms to identify central and bridge symptoms. As far as we know, this study was the first to investigate interconnections among burnout, depression, anxiety, and symptoms of SCL‐90 among Chinese manufacturing employees using network analysis; we submit that our findings will provide a new perspective on the topic and assist in the development of health promotion strategies and illness prevention programs in industrial workplaces.

## 2 Methods

### 2.1 Participants

This study was conducted as a part of the project that aimed to investigate the correlations among employee happiness, mental health, and job characteristics. This study was approved by the Ethics Committee of Guangxi Normal University. All procedures performed in studies involving human participants were in accordance with the ethical standards of the institutional research committee and with the 1964 Helsinki Declaration and its later amendments or comparable ethical standards.

The participants were from an automobile manufacturing company in Sichuan Province, China. All employees in the company were invited to participate. Prior to data collection, we introduced the project to the participants and obtained informed consent from all participants. Data was collected from the company's meeting rooms, and participants were required to fill out the questionnaire via the Wenjuanxing app anonymously and independently. Besides, this study used measures to reduce the influence of common method variance, such as concealing the variable name, mismatching items, and introducing reverse items in advance during the data collection.

There was a total of 5482 employees participating in the study. The part‐time/temporary workers and employees who did not complete the questions were excluded. After the exclusion, 4934 employees were included. The majority of the participants were male (98.1%). The mean age of the participants was 26.8 years (range = 20 to 59 years; SD = 3.76). Of them, 0.4% had junior high school education, 88.1% had senior high school education, and 11.5% had college education or more. Nearly half of the participants (49%) were unmarried.

### 2.2 Measures

The revised Chinese version of the Maslach Burnout Inventory‐General Survey (MBI‐GS) was used to measure job burnout in our study (C. Li and Shi 2003). It comprises 15 items and three subscales: exhaustion (EX, 5 items), cynicism (CY, 4 items), and reduced personal efficacy (PE, 6 items). The participants' responses were rated on a 7‐point Likert scale. The features of PE were reverse scored. Higher scores indicated greater job burnout. In this study, internal consistencies of emotional exhaustion, cynicism, and reduced personal efficacy were 0.93, 0.91, and 0.90, respectively.

The Self‐Rating Anxiety Scale (SAS; Zung 1971) and the Self‐Rating Depression Scale (SDS; Zung 1965) were used to assess anxiety and depression, respectively. Participants' responses were rated on a 4‐point Likert scale. Each scale has 20 items. The sum of the item scores was multiplied by 1.25 to obtain the standard score. Higher standard scores on the SDS or SAS indicate greater levels of mental disorders. In this study, internal consistencies of SAS and SDS were 0.81 and 0.83, respectively.

The Symptom Checklist‐90 (SCL‐90) subscale was used to evaluate employees' psychopathology symptoms (Cheng and Hamid 1996). The seven subscales used in this study were compulsive symptom (10 items), somatization (12 items), psychoticism (10 items), paranoid (6 items), phobic (7 items), hostility (6 items), and interpersonal sensitivity (9 items). Participants' responses were rated on a 5‐point Likert scale. Higher scores for each symptom indicated worse psychological health. In this study, internal consistencies of compulsive symptom, somatization, psychoticism, paranoid, phobic, hostility, and interpersonal sensitivity were 0.82, 0.90, 0.82, 0.78, 0.77, 0.82, and 0.84, respectively.

### 2.3 Analysis

First, we assessed the prevalence of psychological symptoms in manufacturing employees and calculated correlations of the main study variables using SPSS 23.0. Next, we examined the conditional dependence connections between the psychological symptoms using network analysis. The network structure was estimated using the graphical lasso procedure in the R package qgraph (version 1.6.9) (Epskamp et al. 2012). In the network model, symptoms were represented as nodes connected by edges, with the thickness of the edges in the networks indicating the partial correlation coefficients between the nodes. Thicker edges represent stronger relationships and vice versa. The R package (version 1.2.12) was used to estimate the predictability (measured in R2) of the nodes (Haslbeck and Waldorp 2018). The Fruchterman‐Reingold algorithm was used for network visualization, in which the nodes with stronger connections were clustered together (Fruchterman and Reingold 1991). To determine the central nodes in the network, the strength, closeness, and betweenness were calculated. Strength centrality was computed as the sum of all the absolute weights connected to a single node. Betweenness centrality was computed as the number of times that node lies on the shortest path between two other nodes and was used to infer which nodes might frequently act as “middlemen” in network transactions. Closeness centrality was computed as the average distance from a specific node to all other nodes (Valente 2012). The expected influence of a given node is determined by summing all the edge weights between that node and the other nodes in the network; both negative and positive edges surrounding a node were taken into account. Generally, a node with higher centrality interacts with other nodes more strongly and is more vital to the network. Besides, the bridge function in the network tool R package (Jones et al. 2019) was used to calculate bridge expected influence, which is the sum of the edge weights that connect a given node to all those in the other community and is used to determine the bridge nodes in networks with negative and positive connections. Finally, the stability and reliability analysis of the network was assessed by the R package bootnet (version 1.0.5) (Epskamp et al. 2018).

## 3 Results

### 3.1 The Prevalence of Psychological Symptoms in Manufacturing Employees

Table 1 shows the proportion of our samples that experienced burnout, depression, and anxiety. Based on previous studies conducted on Chinese employees (Huo et al. 2021), the total score of each subscale was stratified into high, moderate, or low tertiles as follows: for EX, low EX < 9, moderate EX from 9 to 13, high EX > 13; for CY, low CY < 3, moderate CY from 3 to 9, high CY > 9; and for PE, low PE > 30, moderate PE from 18 to 30, high PE < 18. According to previous research, a high level of EX combined with a high degree of CY or low PE subscale score was diagnosed as burnout, which is called the “exhaustion + 1” criterion (Brenninkmeijer and VanYperen 2003). According to the “exhaustion +1” criterion, 29.77% of the participants met the burnout criteria. The average burnout scores were 11.69 ± 6.48 for EX, 6.99 ± 5.13 for CY, and 21.50 ± 7.79 for PE. We found that 1525 (30.91%), 1217 (24.67%) and 2028 (41.10%) participants experienced high levels of EX, CY, and PE, respectively.

**TABLE 1:** The prevalence of job burnout, depression and anxiety in Chinese manufacturing employee (*n* = 4934).

Variables	*M*	SD	Low	Moderate	High
Exhaustion	11.69	6.48	1673 (33.91%)	1736 (35.18%)	1525 (30.91%)
Cynicism	6.99	5.13	1257 (25.49%)	2460 (49.86%)	1217 (24.67%)
Reduced personal efficacy	14.50	7.79	800 (16.21%)	2106 (42.68%)	2028 (41.10%)
Depression	37.94	7.97	810 (16.44%)	415 (8.42%)	84 (1.70%)
Anxiety	34.01	7.40	795 (16.13%)	202 (4.10%)	46 (0.93%)

In addition, the threshold for the presence of depression (anxiety) was set at a total standard score of 53 (50) points (Feng et al. 2014). Specifically, to define anxiety or depression symptoms, a standardized scoring algorithm was utilized; scores of 50 to 59, 60 to 69, and greater than 69 represented mild, moderate, and severe anxiety, respectively, and scores of 53 to 61, 62 to 71, and greater than 71 respectively represented mild, moderate, and severe depression (Zhang et al. 2022; Zhao et al. 2021). According to the pre‐determined cut‐off points for depression and anxiety, our findings indicated that 1309 (26.53%) participants experienced symptoms of depression, and 1043 (21.14%) participants experienced symptoms of anxiety. In particular, 16.44% (810) of the participants had low levels of depression, while 8.42% (415) and 1.70% (84) had moderate and high levels, respectively. Approximately 16.13% (795) of the participants exhibited low levels of anxiety, while 4.10% (202) and 0.93% (46) had moderate and high levels, respectively. Additionally, we analyzed the prevalence of variables of this study in three age groups. Since the age of most participants in this study was under 30 years (93.8%), there was a similar prevalence across age groups (see Table S1 for more details).

A comparison of the participants' SCL‐90 subscale results and norms (Tong 2010) is shown in Table 2. The seven SCL‐90 features in our sample were found to be significantly higher than the Chinese norms according to the independent sample t‐test we performed (*p <* 0.001). Specifically, compulsive symptom and psychoticism scores in our sample were almost one standard deviation above the norm, marking the greatest divergences, whereas phobia was only slightly above the norm.

**TABLE 2:** Comparison of SCL‐90 subscale results obtained by Chinese manufacturing employees and norm (mean ± SD).

Variables	Manufacturing employee (*n* = 4934)	Norm (*n* = 1890)	*t*
Somatization	1.75 ± 0.63	1.42 ± 0.44	24.40***
Compulsive symptom	2.13 ± 0.57	1.66 ± 0.52	32.52***
Interpersonal sensitivity	1.82 ± 0.57	1.51 ± 0.49	22.32***
Hostility	1.73 ± 0.60	1.50 ± 0.51	15.85***
Phobia	1.40 ± 0.46	1.27 ± 0.39	11.70***
Paranoid	1.73 ± 0.55	1.44 ± 0.47	21.73***
Psychoticism	1.68 ± 0.51	1.33 ± 0.39	30.33***

*** *p* < 0.001.

In addition, the correlations of the main study variables are shown in Table 3. The variables in our study were significantly and positively correlated with each other, which is consistent with previous studies.

**TABLE 3:** Correlations of the main study variables.

	1	2	3	4	5	6	7	8	9	10	11	12
1. EX	1											
2. CY	0.73**	1										
3. PE	0.14**	0.27**	1									
4. DEP	0.51**	0.48**	0.37**	1								
5. ANX	0.55**	0.49**	0.32**	0.68**	1							
6. SOM	0.55**	0.45**	0.16**	0.63**	0.71**	1						
7. COM	0.48**	0.43**	0.17**	0.55**	0.57**	69**	1					
8. SEN	0.43**	0.44**	0.21**	0.54**	0.56**	0.63**	0.77**	1				
9. HOS	0.46**	0.45**	0.20**	0.56**	0.60**	0.63**	0.68**	0.73**	1			
10. PHO	0.35**	0.34**	0.19**	0.49**	0.55**	0.60**	0.63**	0.71**	0.61**	1		
11. PAR	0.43**	0.44**	0.19**	0.51**	0.55**	0.61**	0.71**	0.79**	0.73**	0.66**	1	
12. PSY	0.44**	0.45**	0.18**	0.56**	0.62**	0.70**	0.76**	0.80**	0.71**	0.69**	0.77**	1

Abbreviations: ANX, Anxiety; COM, Compulsive symptom; CY, Cynicism; DEP, Depression; EX, Exhaustion; HOS, Hostility; PAR, Paranoid; PE, Reduced personal efficacy; PHO, Phobia; PSY, Psychoticism; SEN, Interpersonal sensitivity; SOM, Somatization.

** *p* < 0.01.

### 3.2 Network Analysis

**FIGURE 1:**
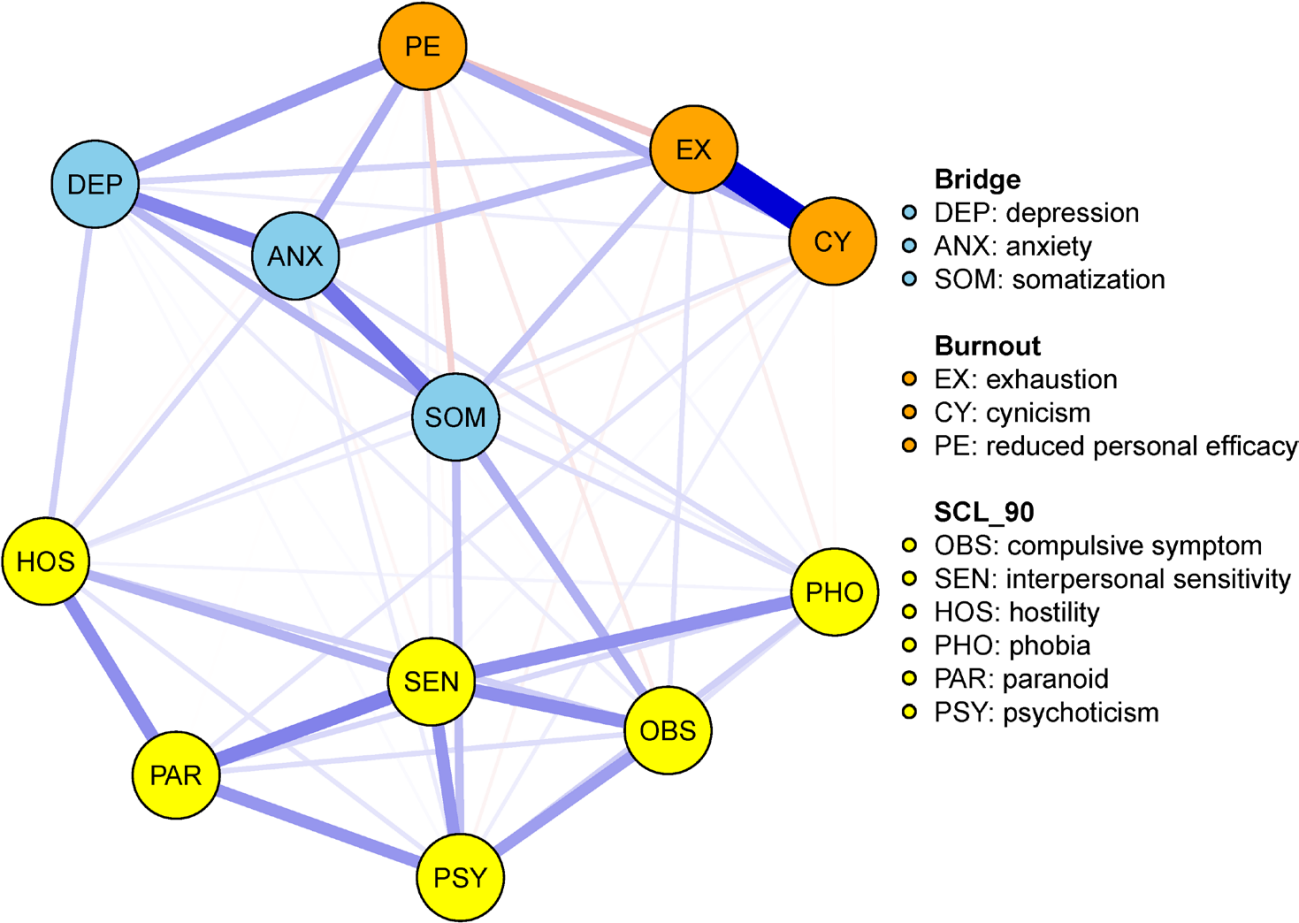
Partial correlation network of exhaustion, cynicism, reduced personal efficacy, depression, anxiety, and SCL‐90 subscales. Blue lines were used to represent positive edges, and red lines were used to represent negative edges. The thicker and more saturated the line, the stronger the connection. Nodes identified as bridge symptoms are colored in blue. Interpersonal sensitivity had the greatest strength, and somatization had the greatest betweenness and closeness. Anxiety had the highest bridge expected influence.

The partial correlation network of exhaustion, cynicism, and reduced personal efficacy, depression, anxiety, and SCL‐90 factors is shown in Figure 1. There were many correlations between the psychological symptoms. The strongest edge was within the two key components of job burnout: ‘exhaustion – cynicism’ (weight = 0.563), which is consist with previous studies (Tao et al. 2024; von Känel et al. 2020). The correlations between EX, CY, PE, and the remaining symptoms differed. Specifically, EX was not related to interpersonal sensitivity, hostility, or paranoid ideation, and connected strongly to anxiety (weight = 0.153) and somatization (weight = 0.128). CY was not related to anxiety or obsessive‐compulsive symptoms and connected to hostility strongly (weight = 0.063). PE was correlated with all other symptoms and connected strongly to depression (weight = 0.222) and anxiety (weight = 0.178). In addition, connections among the three components of burnout, depression, and anxiety were stronger than that of the symptoms of SCL‐90. Except for paranoid ideation, depression and anxiety were related to the remaining symptoms of the SCL‐90, and the connections among the symptoms of the SCL‐90 were strong. Somatization was more closely linked to anxiety and depression than other symptoms of the SCL‐90. More importantly, the strongest intergroup association was ‘somatization‐anxiety’ (weight = 0.305). Somatization connected strongly to anxiety, suggesting the physical and anxiety symptoms intermingle.

Additionally, the predictability of interpersonal sensitivity was the highest (see Table S2 for more details).

**FIGURE 2:**
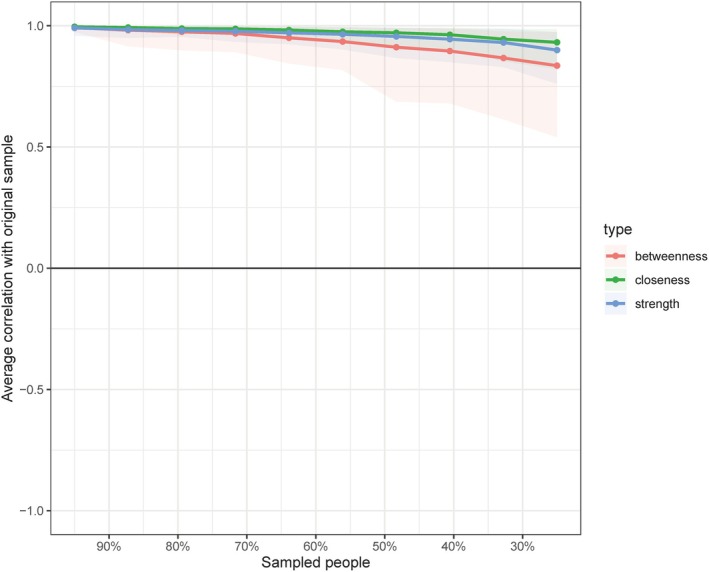
Case dropping bootstrap for the network shown in Figure 1. The dots represent the average correlation between centrality indices of the original network and the bootstrapped networks consisting of a subset of participants (with decreasing sample size from left to right).

Table 4 shows the standardized centrality and bridging of the nodes in the network, namely strength, betweenness, closeness, and bridge expected influence. Centrality stability was assessed using the correlation stability coefficient (CS‐coefficient) which represents the maximum proportion of the original sample that can be dropped while still maintaining a correlation of 0.7 with the original centrality indices (Epskamp et al. 2018). Since centrality indices with a CS‐coefficient greater than 0.25 (preferentially greater than 0.5) are considered interpretable, we then calculated the CS‐coefficient to assess centrality stability (Figure 2). In this study, the CS for strength was 0.750, betweenness was 0.594, closeness was 0.750, and bridge expected influence was 0.70. Therefore, the ranking of the symptoms that the centrality indices quantified in this study should be considered strongly robust and trustworthy. Our results showed that interpersonal sensitivity (Z_strength_ = 1.576) had the greatest strength, followed in order by exhaustion, anxiety, and somatization. Somatization had the highest scores for both closeness (Z_closeness_ = 1.506) and betweenness (Z_betweenness_ = 2.453). In terms of bridge symptoms, anxiety had the highest bridge expected influence (Z_bridge expected influence_ = 1.718), followed in order by depression and somatization. These results indicated that interpersonal sensitivity and somatization emerged as the core symptoms, and anxiety was an important bridge symptom in the health network of Chinese manufacturing workers.

**TABLE 4:** Centrality and bridge indices of each node depicted in the network.

Variables	Betweenness	Closeness	Strength	Bridge expected influence
Exhaustion	0.250	−0.986	1.054	0.748
Cynicism	−0.751	−1.332	−0.654	0.584
Reduced personal efficacy	−0.751	−0.796	−1.191	0.425
Depression	−0.551	−0.036	−0.636	1.609
Anxiety	1.051	1.373	0.922	1.718
Somatization	2.453	1.506	0.781	0.808
Compulsive symptom	0.651	1.137	−0.004	0.239
Interpersonal sensitivity	0.250	0.326	1.576	0.139
Hostility	−0.551	−0.486	−0.583	0.447
Phobia	−0.951	−1.142	−1.782	0.195
Paranoid	−0.751	−0.249	0.104	0.188
Psychoticism	−0.350	0.683	0.413	0.223

*Note:* The indices were shown as standardized values *z*‐scores.

Finally, we utilized the bootnet package to assess the accuracy and stability of the network. Specifically, we used a bootstrap approach (1000 bootstrap samples) to calculate 95% confidence intervals (CI) for the edge weights and performed tests to determine the difference in edge weights using bootstrapped difference tests (1000 bootstrap samples). The results showed that the accuracy and stability of the network in this study were robust (Figures S1 and S2 for more details).

## 4 Discussion

This study, as far as we know, was the first to utilize network analysis to explore the prevalence and association of burnout, depression, anxiety, and SCL‐90 in Chinese manufacturing employees. In total, we found that: (1) 29.77%, 21.14%, and 26.53% of all participants experienced burnout, anxiety, and depression, respectively; (2) the symptoms of the SCL‐90 were significantly higher among participants compared to normative data of the Chinese population; (3) anxiety was the important bridge symptom within the health network; (4) interpersonal sensitivity and somatization emerged as the core symptoms. The following sections will discuss these findings.

Job burnout occurs in individuals who are exposed to chronic stressors for a prolonged period of time, which is harmful to both the individuals who experience it and to society as a whole (Maslach et al. 2001). Our results show that 29.77% of the participants experienced job burnout. The prevalence observed in manufacturing employees is lower than that in other Chinese occupational subgroups, such as physicians in intensive care units (Wang et al. 2021), frontline healthcare workers (Zhang et al. 2021), and preschool teachers (Li et al. 2020). This is, however, higher than the prevalence among village doctors (Zhao et al. 2021) and medical staff in Liaoning province, China (Guo et al. 2021). It is crucial to highlight that the results across research differ not only as a consequence of different assessment tools for burnout being utilized, but also due to the fact that different criteria are used to diagnose burnout. Our study follows the “exhaustion + 1” criterion, which means that an individual may be deeded to suffering from burnout when the individual reports a high level of emotional exhaustion in combination with a high level of cynicism or a low level of personal efficacy (Brenninkmeijer and VanYperen 2003). This criterion used in this study is consistent with the notion that exhaustion is a crucial indicator of burnout, and this is supported by the fact that the only dimension contained in any of the burnout diagnostic tools is exhaustion (Huo et al. 2021). Additionally, considering the different criteria used to diagnose burnout, we directly contrasted the specific burnout scores across studies that used the same scale. Compared with the various studies conducted in China, we found that the exhaustion and cynicism scores of manufacturing employees were comparable to those of medical staff and other high‐risk groups (Wu et al. 2013, 2014). The manufacturing employees' score for personal efficacy was even higher than that of healthcare workers (He et al. 2019; Huo et al. 2021), suggesting that burnout is a significant problem among workers in the manufacturing sector.

Regarding the prevalence of depression and anxiety, our results showed that 26.53% and 21.14% of the participants in our sample experienced symptoms of depression and anxiety, respectively. Earlier research has indicated that globally, the 12‐month prevalence of anxiety disorders ranges from 2.4% to 18.2%, with a pooled 1‐year prevalence rate of 10.6% and a lifetime prevalence rate of 16.6% (Battams et al. 2014; Somers et al. 2006). Similarly, Roche et al. (2016) reviewed the studies on male‐dominated sectors and found that the reported prevalence rates of depression among workers in the manufacturing industry ranged from 2.6% to 23.4%. This suggests that there is a high prevalence rate of anxiety and depression among manufacturing workers in China. Besides, the prevalence of anxiety and depression among Chinese manufacturing workers in this study was higher than that in other Chinese subgroups, such as individuals suffering from chronic obstructive pulmonary disease (Xiao et al. 2018) and medical students (Xiao et al. 2020). Additionally, compared to Chinese adult normative scores, the seven symptoms of the SCL‐90 among the participants were significantly higher, indicating that manufacturing employees generally had certain psychological symptoms. In summary, these findings indicate that the status of Chinese manufacturing employees' mental health is concerning and requires appropriate intervention.

Besides, the network analysis revealed a dense network structure, suggesting that there were many correlations between burnout, depression, anxiety, and SCL‐90 factors. The three burnout dimensions were connected to almost all the other symptoms, and their correlations with depression and anxiety were stronger than their connections with the symptoms of the SCL‐90. These diverse and unique relationships are consistent with previous studies and provide further evidence that burnout as an occupational psychological syndrome is related to many mental health difficulties and is closely related to anxiety and depression (Ernst et al. 2021; Salvagioni et al. 2017).

Moreover, the network analysis showed that the strongest intergroup association was between anxiety and somatization, and anxiety was an important bridge symptom within the health network, suggesting the physical and anxiety symptoms intermingle. Studies revealed high comorbidity rates, with over 50% of cases exhibiting overlap between somatic symptoms and anxiety (Löwe et al. 2008). In addition, anxiety, depression, and somatization might function as bridge symptoms connecting burnout with other syndromes, which is consistent with previous studies suggesting shared psychobiological mechanisms underlying these conditions (Stein and Muller 2008).

Furthermore, the analysis of the nodes' centrality indicated that somatization and interpersonal sensitivity were the core symptoms within the health network of Chinese manufacturing employees. Specifically, somatization is defined as somatoform bodily complaints and related to a variety of physical symptoms, such as dizziness, musculoskeletal complaints and fatigue (Shangguan et al. 2021). It seems that in Chinese manufacturing settings, workers may somaticize stress (present with headaches, fatigue, etc.). It is not just a random finding, but one aligned with cultural expectations. The cultural psychology studies reported that Chinese (and East Asian) individuals often report somatic symptoms for psychological distress, sometimes more readily than emotional symptoms, as a culturally shaped pattern (Ryder and Chentsova‐Dutton 2012; Zhou et al. 2016). This also would explain why somatization is so prominent in the network and indicate that somatic complaints might be an entry point for interventions among Chinese manufacturing employees. The other potential explanation is that due to the nature of the manufacturing industry, most participants in our study are male. A growing body of research on health‐related help‐seeking behavior indicates that because of the stigma related to mental illness and conventional masculinity norms, men are less likely than women to acknowledge that they need help or seek help for mental disorder problems. This view coupled with the fact that when they do become ill, men's psychological illness more often than not manifests as a subjective sense of physical discomfort, may explain why somatization is an important symptom in the network of manufacturing employees (Battams et al. 2014). In addition, interpersonal sensitivity, a personality trait linked to anxious attachment, is closely associated with negative core beliefs about the self, suggesting it may be a cognitive vulnerability to depression (Otani et al. 2018). A previous study found that interpersonal sensitivity had been closely linked to poor psychological functioning (Salvagioni et al. 2017). Our results are in line with those of earlier research and further highlight the importance of somatization and interpersonal sensitivity in the association of psychological symptoms among Chinese manufacturing employees.

Our findings may provide several implications for mental healthcare providers and firm administrators. Firstly, given the high prevalence of psychological symptoms in manufacturing employees, we submit that a systematic assessment of the mental health of this at‐risk group needs to be undertaken and mental health services should be provided. Secondly, given the close relationship between burnout, depression, anxiety, and SCL‐90 factors, such assessment should not be restricted to burnout, but should also include screening for symptoms of anxiety and depression as well as symptoms of the SCL‐90. Thirdly, the finding that somatization and interpersonal sensitivity are highly central symptoms suggests that interventions to reduce chronic fatigue (e.g., through workload management or rest breaks) and to improve the interpersonal work environment (e.g., conflict resolution, team support training) might have broad positive effects on workers' mental health. Besides, interventions teaching stress management or emotional awareness might reduce the need for somatic expression and thereby improve multiple outcomes. More importantly, Chinese workers may be more likely to seek help for physical complaints, so medical staff in factories should be alert that these complaints may mask underlying psychological issues and screening for physical symptoms in employee health check‐ups could be an effective strategy to identify at‐risk individuals. Mental health promotion activities should also be considered in mental health services to help employees relax, such as training for improving resilience, mindfulness practicing lessons, yoga classes, and so on.

This study had several limitations. Firstly, the connections between symptoms did not infer causality since the data used in this study was cross‐sectional. Secondly, our participants in this study only comprised employees who worked in the manufacturing sector. As a result, caution is advised when applying the findings of this study to other subgroups. Moreover, due to the nature of the manufacturing industry, most participants in our study were male, which may limit the results' application to the female population. Besides, since factors such as length of employment were not collected in our survey, we cannot examine whether employment duration can influence the mental health network. Future research should be conducted by collecting more information about these factors. Furthermore, the assessment of psychological symptoms in this study was based on self‐reporting only, which might have led to an overestimation or underestimation of symptoms compared to what would have been assessed in clinical interviews. Thus, when interpreting the findings of this study in this regard, caution is advised.

In summary, this study found that there was a high prevalence rate of burnout, depression, and anxiety among Chinese manufacturing employees, and the symptoms of the SCL‐90 were significantly higher in this subgroup than Chinese norms. Furthermore, the network analysis revealed many connections between the psychological symptoms. Interpersonal sensitivity and somatization emerged as the core symptoms, and anxiety was an important bridge symptom. Interventions aimed at these conditions may promote and enhance the overall mental health of Chinese manufacturing employees.

## Conflicts of Interest

The authors declare no conflicts of interest.

## Supporting information


**DATA S1.** Supporting Information.
