# Supplementary material for: Psychological Distress Among Chinese Manufacturing Employees: Prevalence and a Symptom Network Analysis
Source: Psych J. 2025 Apr 29;14(4):603–13. doi: 10.1002/pchj.70015 (PMC12318592; doi:10.1002/pchj.70015)
Supplement: Supplementary file 1 — DATA S1. Supporting Information. [file PCHJ-14-603-s001.docx]

SUPPLEMENTARY

Table S1. the prevalence of job burnout, depression and anxiety in different age groups

|  | age 20-25 (n=1641) | | |  | age 26-30(n=2985) | | |  | age 31-59(n=308) | | |
| --- | --- | --- | --- | --- | --- | --- | --- | --- | --- | --- | --- |
| Variables | low | mid | high |  | low | mid | high |  | low | mid | high |
| EX | 558(34%) | 582(35.5%) | 501(30.5%) |  | 931(31.2%) | 1064(35.6%) | 990(33.2%) |  | 184(59.7%) | 90(29.2%) | 34(11%) |
| CY | 381(23.2%) | 860(52.4%) | 400(24.4%) |  | 462(15.5%) | 1733(58%) | 790(26.5%) |  | 98(31.8%) | 183(59.4%) | 27(8.8%) |
| PE | 24(1.4%) | 652(39.8%) | 965(58.8%) |  | 59(2%) | 1189(39.8%) | 1737(58.2%) |  | 9(2.9%) | 100(32.5%) | 199(64.6%) |
| Depression | 237(14.4%) | 137(8.3%) | 19(1.2%） |  | 536(18%) | 258(8.6%) | 62（2.1%） |  | 37(12%) | 20(6.5%) | 3(1%) |
| Anxiety | 269(16.4%) | 57(3.5%) | 11(0.7%) |  | 487(16.3%) | 141(4.7%) | 35(1.2%) |  | 39(12.7%) | 4(1.3%) | 0 |

*Note.* EX = exhaustion, CY = cynicism, PE = reduced personal efficacy

| Node | Predictability |
| --- | --- |
| Exhaustion | 0.600 |
| Cynicism | 0.563 |
| Reduced personal efficacy | 0.224 |
| Depression | 0.562 |
| Anxiety | 0.617 |
| Somatization | 0.644 |
| Compulsive symptom | 0.694 |
| Interpersonal sensitivity | 0.763 |
| Hostility | 0.641 |
| Phobia | 0.550 |
| Paranoid | 0.705 |
| Psychoticism | 0.726 |

Table S2. Predictability of the nodes for the network depictured in Figure 1


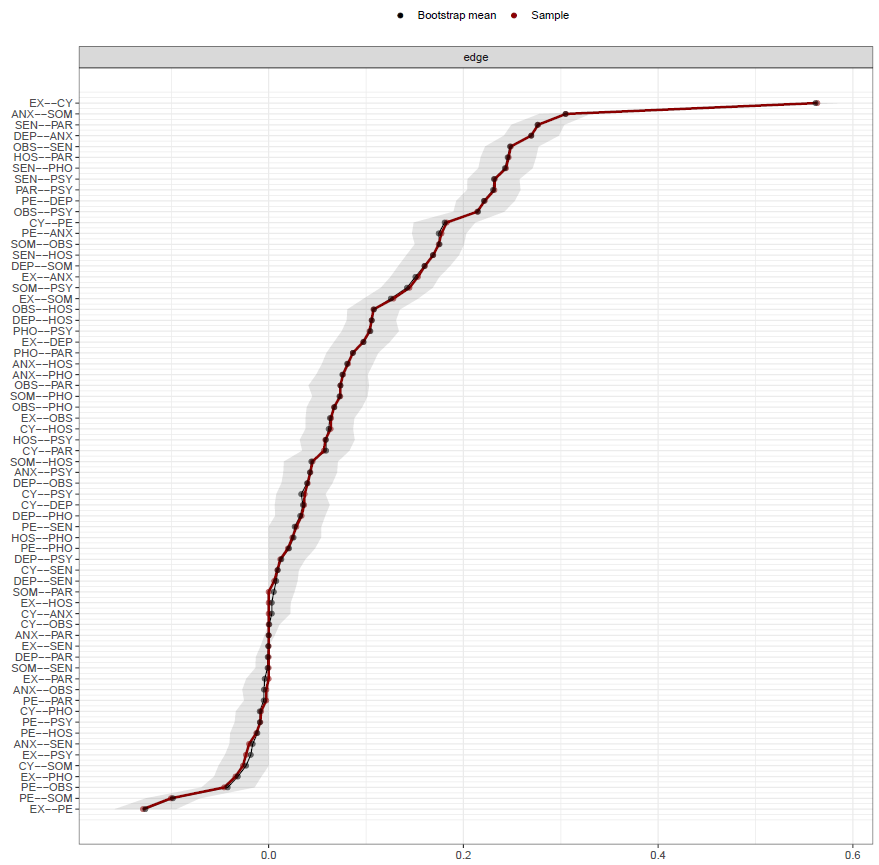


Figure S1. Bootstrap 95% confidence intervals of the edge weights of the network shown in figure 1. Individual edges are represented by a horizontal grey line, the edges’ weight is indicated by the red line. The grey area indicates the bootstrapped 95% confidence interval. EX = exhaustion, CY = cynicism, PE = reduced personal efficacy, DEP = depression, ANX = anxiety, SOM = somatization, OBS = compulsive symptom, SEN = interpersonal sensitivity, HOS = hostility, PHO = phobia, PAR = paranoid, PSY = psychoticism.


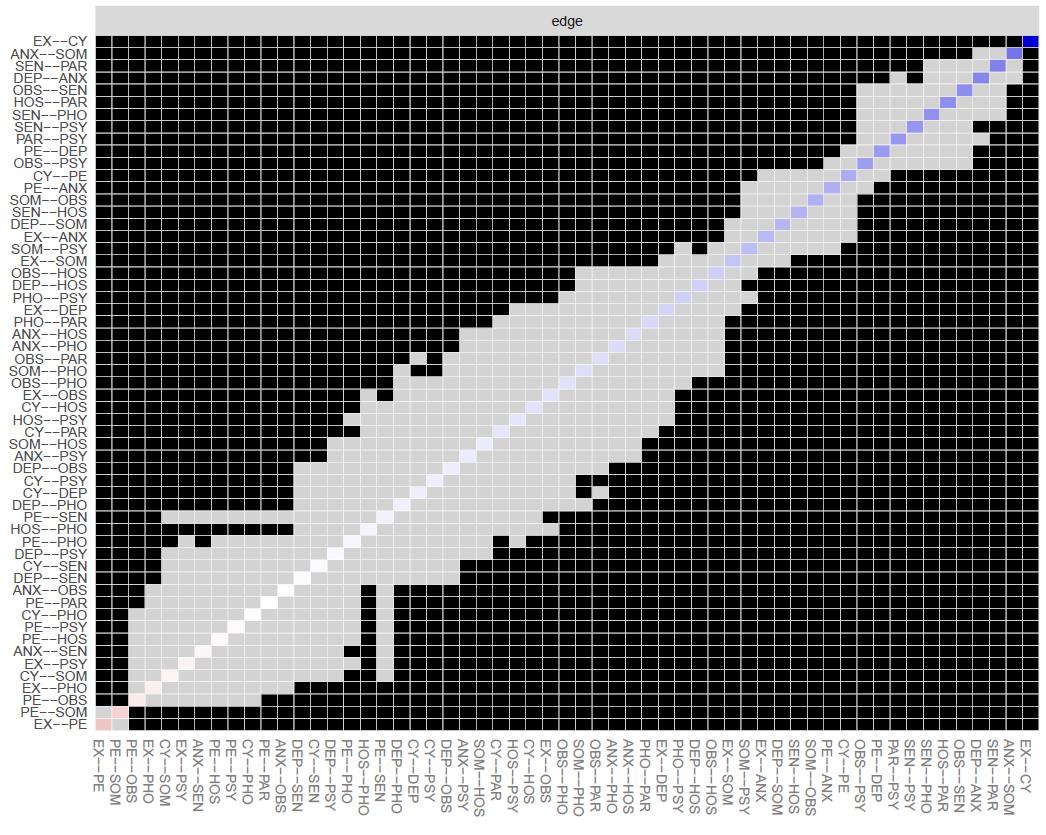


Figure S2. Bootstrap edge weights difference test between edge-weights in the network shown in Figure 1. Grey-colored boxes represent non-significant differences, while black boxes represent significant differences. The diagonal line indicates the strength of edge-weights, shifting from red (negative associations), to white (representing weaker edges) and ultimately dark blue (representing stronger edge-weights). EX = exhaustion, CY = cynicism, PE = reduced personal efficacy, DEP = depression, ANX = anxiety, SOM = somatization, OBS = compulsive symptom, SEN = interpersonal sensitivity, HOS = hostility, PHO = phobia, PAR = paranoid, PSY = psychoticism.
